# Music Attuned Technology: Care via eHealth (MATCH): A proof‐of‐concept study trialling a music therapy informed mobile application for caregivers of people living with dementia

**DOI:** 10.1002/alz.094181

**Published:** 2025-01-09

**Authors:** Felicity Baker, Tanara Sousa, Jeanette Tamplin, Jenny Waycott, Zara Thompson, Dianna Vidas, Robyn Woodward‐Kron, Phoebe Stretton‐Smith, Nicola T Lautenschlager, Amit Lampit, Lars Kulik, Stan Skafidas

**Affiliations:** ^1^ The University of Melbourne, Melbourne, VIC Australia; ^2^ Faculty of Fine Arts and Music, The University of Melbourne, Melbourne, VIC Australia; ^3^ The University of Melbourne, Parkville, VIC Australia

## Abstract

**Background:**

MATCH (Music Attuned Technology – Care via eHealth) is a music and health application that supports caregivers of people living with dementia to use music strategically to better manage care through virtual training and intuitive music technology. This study trialled a prototype version of the MATCH app with family caregivers and people with dementia residing in the community.

**Method:**

16 Dyads trialled the prototype MATCH app. After completing the virtual training in the app, family caregivers created personalised playlists within the app and used strategies learnt during training with the person they care for at least twice per week over 8 weeks. Pre‐post measures on the Neuropsychiatric Inventory Questionnaire (NPI‐Q), Cohen‐Mansfield Agitation Inventory and Acceptability of the mobile app and training were captured to detect change in symptoms and feasibility of the application. Qualitative measures including a participation diary and qualitative interviews captured participant perspectives and usage of the app.

**Results:**

Significant pre‐post changes in symptom severity (‐4.23 [4.9], 95% CI ‐7.2 to ‐1.3, p = 0.009) and symptom distress (‐5.69 [5.8], 95% CI ‐9.2 to ‐2.2, p = 0.004) on the NPI‐Q were found, but not on the Cohen‐Mansfield Agitation Inventory (‐4.38 [11.6], 95% CI ‐9.9 to 1.34, p = 0.109). Subscales of the NPI‐Q found significant changes to mood symptoms (‐1.23 [1.9], 95% CI ‐2.36 to ‐0.01, p = 0.036) and frontal symptoms (‐1.45 [23], 95% CI ‐2.87 to ‐0.05, p = 0.043), but not agitation/aggression (‐1.1 (1.9], 95% CI ‐2.21 to 0.07, p = 0.062). 85% of carers were satisfied with the MATCH app features, and 92% were satisfied with the music training. Qualitative findings revealed that participants found the strategies to be effective, and that they were able to integrate music‐based strategies into their care routines.

**Conclusion:**

The MATCH app was found to be feasible and acceptable; family caregivers reported that MATCH training was understandable and useful. There is preliminary evidence that music interventions implemented by trained family caregivers are effective in reducing neuropsychiatric symptoms.

